# Identification of Oxidative-Stress-Reducing Plant Extracts from a Novel Extract Library—Comparative Analysis of Cell-Free and Cell-Based In Vitro Assays to Quantitate Antioxidant Activity

**DOI:** 10.3390/antiox13030297

**Published:** 2024-02-28

**Authors:** Mara Heckmann, Verena Stadlbauer, Ivana Drotarova, Theresa Gramatte, Michaela Feichtinger, Verena Arnaut, Stefanie Atzmüller, Bettina Schwarzinger, Clemens Röhrl, Bernhard Blank-Landeshammer, Julian Weghuber

**Affiliations:** 1 Center of Excellence Food Technology and Nutrition, University of Applied Sciences Upper Austria, Stelzhamerstraße 23, 4600 Wels, Austria; mara.heckmann@fh-wels.at (M.H.); verena.stadlbauer@fh-wels.at (V.S.); ivana.drotarova@fh-wels.at (I.D.); theresa.gramatte@fh-wels.at (T.G.); michaela.feichtinger@fh-wels.at (M.F.); verena.arnaut@fh-wels.at (V.A.); stefanie.atzmueller@fh-wels.at (S.A.); bettina.schwarzinger@fh-wels.at (B.S.); clemens.roehrl@fh-wels.at (C.R.); bernhard.blank-landeshammer@fh-wels.at (B.B.-L.); 2 FFoQSI GmbH–Austrian Competence Centre for Feed and Food Quality, Safety and Innovation, Technopark 1D, 3430 Tulln, Austria

**Keywords:** oxidative stress, plant extracts, antioxidant, extract library, screening, ROS, NO

## Abstract

Numerous underexplored plant species are believed to possess considerable potential in combating oxidative stress and its associated health impacts, emphasizing the need for a comprehensive methodological screening approach to assess their antioxidant capacity. This study investigated 375 plant extracts, utilizing both cell-free and cellular methods to evaluate their antioxidant properties. Target-based antioxidant capacity was evaluated by the total phenolic content (TPC) and ferric reducing antioxidant power (FRAP) assays. Cell-based assays employed the H_2_DCF-DA probe to measure reactive oxygen species (ROS) levels and the Griess assay to quantify nitric oxide (NO) levels in stressed Caco-2 and RAW264.7 cells, respectively. The highest TPC and FRAP values were found in extracts of *Origanum vulgare* and *Fragaria × ananassa* leaves. Several plant extracts significantly reduced stress-induced ROS or NO levels by at least 30%. Distinctive selectivity was noted in certain extracts, favoring the significant reduction of NO (e.g., *Helianthus tuberosus* extract), of ROS (e.g., *Prunus domestica* subsp. *Syriaca* extract), or of both (e.g., *Fragaria × ananassa leaf* extract). A strong correlation between TPC and FRAP values and moderate correlations between the results of the cell-free and cell-based assays were evident. These findings highlight the great antioxidant potential of underexplored plant extracts and the diversity of the underlying mechanisms, emphasizing the importance of a multifaceted approach for a comprehensive assessment.

## 1. Introduction

Plants have historically served as a repertoire of bioactive compounds used in traditional medicine across cultures. The diversity of bioactive compounds present in plant sources has maintained scientific interest in their potential therapeutic applications [1]. Through the investigation of plant-derived antioxidants, a spectrum of compounds including polyphenols, flavonoids, carotenoids, and various vitamins were identified. These bioactive constituents exhibit remarkable abilities to mitigate oxidative stress by neutralizing free radicals, a process closely associated with several chronic diseases and ageing [2].

Oxidative stress, driven by the excessive generation of reactive oxygen species (ROS) and reactive nitrogen species (RNS) such as nitric oxide (NO), poses a significant challenge to cellular integrity. ROS, which include molecules such as the superoxide anion, hydrogen peroxide, and the hydroxyl anion, along with NO, cause damaging effects when produced in excess [3]. These reactive species contribute to lipid peroxidation, protein oxidation, and DNA damage, thereby disrupting cellular function. While moderate levels of NO and ROS play an essential role in cell signaling and immune responses by enhancing the clearance of pathogens and damaged cells, overproduction and dysregulation can promote the progression of several chronic diseases, including diabetes, atherosclerosis, or inflammatory bowel disease [3,4,5]. Plant-derived bioactive compounds such as the polyphenols resveratrol from grapes and quercetin from onions show strong ROS and NO scavenging properties [2]. Similarly, carotenoids such as lycopene and β-carotene exhibit potent antioxidant activity, effectively scavenging ROS and RNS to mitigate oxidative damage [6,7]. Moreover, compounds such as catechins from green tea and proanthocyanidins found in grape seeds have been particularly noted for their potent antioxidant properties and putative derived health benefits [8,9].

The effective evaluation of the antioxidant capacity of plant extracts involves a diverse range of assays for adopting a comprehensive approach for activity assessment. Commonly used cell-free assays to determine the antioxidant potential of plant extracts include total phenolic compound content (TPC) and ferric reducing antioxidant power (FRAP) assays [10,11,12]. The TPC assay provides a fast quantification of the total phenolic content of a sample, which directly correlates with its antioxidant capacity due to the well-established antioxidant properties inherent in phenolic compounds [13,14,15,16]. This assay is straightforward, cost-effective, and widely applicable. However, it is important to note that, while TPC estimates the potential antioxidant capacity based on the phenolic content, it might not fully capture other non-phenolic antioxidants present in the extract [17]. On the other hand, the FRAP assay measures the ability of antioxidants to reduce ferric ions to ferrous ions in a redox-linked colorimetric reaction. FRAP directly assesses the ability of antioxidants to reduce oxidative species [18]. This assay is sensitive and rapid, and enables a straightforward comparison of the antioxidant capacity between different samples. Yet, FRAP measures a specific type of antioxidant activity (reducing power) and might not fully reflect the overall antioxidant potential of the extract, especially considering other mechanisms involved in the antioxidant process [19].

To forge a connection between target-based, cell-free analysis and biological significance, cell-based assays are often additionally utilized for the assessment of the antioxidant potential [20,21]. While cell-free assays provide a controlled and simplified environment for a quick initial assessment of the antioxidant capacity, they lack the ability to account for the complex cellular environment. This limitation highlights the importance of cellular assays that consider critical factors such as uptake, metabolism, and cellular interactions and signaling. Such assessments provide a better understanding of how plant extracts interact with cellular components, affect signaling cascades, and potentially influence gene expression associated with oxidative stress responses [21]. Two examples of cellular assays, which target different aspects of oxidative stress, include the ROS detection assay utilizing 2′,7′-dichlorodihydrofluorescein diacetate (H_2_DCF-DA) [12,22,23], and the Griess assay [24]. This ROS detection assay is based on the principle of detecting ROS-induced oxidative stress. H_2_DCF-DA, a cell-permeable dye, is oxidized by ROS to produce a fluorescent compound. This assay is particularly useful for directly measuring the ability of a sample to scavenge or neutralize ROS in a biological context. It is commonly used in cellular studies and provides a real-time assessment of antioxidant activity and mitigation of ROS-induced damage [25]. In contrast, the Griess assay focuses on the detection of the RNS NO. This assay quantifies the levels of nitrite (NO_2−_), a stable oxidation product of NO, and is suitable for assessing the NO scavenging activity of antioxidant plant extracts and compounds [26]. The Griess assay is versatile and can be applied across various biological samples, aiding in the understanding of the impact of antioxidants on NO-mediated oxidative stress [24,27,28]. In biological systems, inflammatory processes often intertwine with oxidative stress mechanisms, creating a complex relationship that lacks a clear-cut boundary. Excessive ROS and RNS production can contribute to inflammatory responses and vice versa [29]. Integrating these assays provides a more comprehensive understanding on the antioxidant capacity of bioactive compounds and their potential impact on inflammatory pathways.

Despite the notable potential of plant-based reagents and extracts to counteract oxidative stress and its adverse health effects, a substantial proportion of edible plant species remain underexplored. This suggests a significant reservoir of antioxidant-rich compounds that have yet to be thoroughly investigated and utilized for their potential therapeutic applications [30,31]. At the same time, the food industry has witnessed a pronounced shift towards functional foods and dietary supplements, along with a surge in the use of clean label and regional products [32]. With its implementation in 2014, the Nagoya Protocol on Access and Benefit Sharing marked a significant milestone in international biodiversity governance, highlighting the need for responsible and sustainable use of regional biodiversity and local genetic resources [33]. This shift has opened new avenues for the identification and screening of edible, locally grown plants, particularly in the context of their bioactive properties. Although various large plant extract libraries have been collected and curated to date [34,35,36,37,38], a significant portion remains inaccessible to the public [39] or consists of raw materials sourced globally [34]. An additional barrier for the use of these libraries, particularly for potential manufacturers committed to the Nagoya Protocol on Access and Benefit Sharing, is the lack of documentation regarding the origin of these botanical raw materials [33].

In this study, we used a plant extract library that comprises 375 individual extracts prepared from mostly edible plants collected primarily in Upper Austria and bordering regions. The aim of this research was to screen this library for potent antioxidant properties utilizing a dual approach by employing both cell-free (TPC and FRAP) and cell-based (ROS and NO detection) methods. Our objective was to comprehensively evaluate the antioxidant potential of these extracts. By comparing the potential of these different assays, we aimed to elucidate their respective strengths for a large-scale assessment of antioxidant capacity. In addition, we examined the correlation between these methods to uncover potential associations or discrepancies in the evaluation of the antioxidant properties of the screened plant extracts.

## 2. Materials and Methods

### 2.1. Plant Sample Collection, Categorization, and Pretreatment

Plant raw materials were collected and identified using the Flora Incognita app [40]. Selection for the extract library followed specific criteria: (1) local growth in Upper Austria, (2) non-toxic nature with a history of safe use of at least one plant part, and (3) absence from the endangered list of the Upper Austrian Red List of vascular plants [41].

Collection details were documented; the material was transported to the laboratory for photography and immediate pre-treatment. A gentle drying process was then applied: crushed herbs and spices were dried overnight in a drying cabinet at 50 °C, while cut fruits, vegetables, and mushrooms were dried in a dehydrator for 18–48 h. This was followed by grinding in a commercial coffee grinder. Processing varied depending on the plant material available—individual parts or pools were treated accordingly. The ground material was further photographed, and dry matter was determined using a moisture analyzer (MA 160 Moisture Analyzer, Sartorius, Göttingen, Germany). Samples of 0.5 g were heated to 105 °C until complete dryness.

### 2.2. Plant Extract Preparation

The plant extracts were prepared using the following method: 5 g of dried ground raw material were added to 30 mL of hot water at 70 °C, and vortexed for 1–2 min to achieve uniform saturation, then placed on an overhead shaker for 10 min. After centrifugation at 4149 rcf for 10 min, the supernatant was removed. The residue was mixed twice with approximately 10 mL of hot water, then vortexed and centrifuged as described above. The combined supernatants were filtered to a final volume of 50 mL. pH and dry matter content were determined using a moisture analyzer (MA 160 Moisture Analyzer, Sartorius, Göttingen, Germany) at 110 °C on 2 g of extract. Aliquoted extracts were stored at −80 °C until further use.

### 2.3. Determination of Total Phenolic Content

Total phenolic content (TPC) was determined using gallic acid as a reference standard [42]. Prior to use, the extract samples were centrifuged at 17,000 rcf for 10 min. Subsequently, 100 µL of the extract was mixed with 1 mL of distilled water and 100 µL of Folin–Ciocalteu reagent from (Sigma-Aldrich, St. Louis, MO, USA). After 6 min of incubation at room temperature, 500 µL of saturated sodium carbonate solution (Sigma-Aldrich) was added to the sample, followed by a 70–75-min incubation in the dark at room temperature. Then, 200 µL of the sample mixtures, along with blanks and standards, were pipetted into a 96-well plate in triplicate. The plate was incubated for 75 min in the dark at room temperature. After incubation, absorbance was measured at 750 nm using a microplate reader (POLARstar Omega, BMG Labtech, Ortenberg, Germany). The results were expressed as gallic acid equivalents (GAE) per liter plant extract.

### 2.4. Determination of Ferric Reducing Antioxidant Power

Total antioxidant activity was measured by the ferric reducing antioxidant power (FRAP) assay as described previously [43], using (±)-6-hydroxy-2,5,7,8-tetramethylchroman-2-carboxylic acid (TROLOX) as a reference standard. Briefly, the FRAP solution was prepared by mixing 300 mM acetate buffer (pH 3.6), 10 mM 2, 4, 6-tripyridyl-s- triazine (TPTZ), diluted in 40 mM hydrochloride acid and 20 mM ferric chloride in a ratio of 10:1:1, respectively. All the reagents were obtained from Sigma-Aldrich (St. Louis, MO, USA). Subsequently, 6 µL of the extract samples, along with blanks and standards, were mixed with 180 µL of the FRAP solution (37 °C) and pipetted into a 96-well plate in triplicate. Absorbance was measured at 593 nm immediately and every minute until the reactions stopped completely, as indicated by no changes in absorption values, using the POLARstar Omega plate reader (BMG Labtech). The results were expressed as TROLOX equivalents (TE) per liter plant extract.

### 2.5. Cell Culture

RAW264.7 mouse macrophage-like (ATCC, Manassas, VA, USA) and Caco-2 human epithelial (DSMZ, Braunschweig, Germany) cell lines were cultured under conditions of 37 °C, 5% CO_2_, and humidified air in Dulbecco’s Modified Eagle Medium (DMEM, +4.5 g/L glucose, +stable glutamine, +sodium pyruvate. +3.7 g/L sodium bicarbonate) and Eagle Minimum Essential Medium (EMEM, +earle’s balanced salts, +2 mM L-glutamine, +non-essential amino acid, +1 mM sodium pyruvate, +1.5 g/L sodium bicarbonate), respectively, supplemented with 10% fetal bovine serum (FBS) and 1% penicillin-streptomycin (P/S) (all from PAN-Biotech, Aidenbach, Germany). The cells were subcultured two times a week at splitting ratios between 1:4 and 1:10, depending on the confluency. For this, Caco-2 cells were washed with phosphate-buffered saline (PBS) without calcium and magnesium (PAN-Biotech), detached using trypsin-EDTA solution (Biochrom GmbH, Berlin, Germany) and neutralized in fresh EMEM. RAW264.7 cells were detached with a cell scraper (TPP Techno Plastic Products AG, Trasadingen, Switzerland) and suspended in fresh DMEM.

### 2.6. Detection of ROS Production in Caco-2 Cells

Intracellular ROS production was quantified via the cell-permeant probe H_2_DCF-DA following stress induction by the free-radical-generating compound 2,2′-Azobis(2-amidinopropane) dihydrochloride (AAPH) in living Caco-2 cells. Once inside the cell, this probe undergoes enzymatic cleavage and is converted to a non-fluorescent state. In the presence of ROS molecules, this transformed probe is oxidized to produce the highly fluorescent compound 2′,7′-dichlorofluorescein (DCF). The increase in fluorescence intensity directly corresponds to the amount of ROS generated within the cells, allowing for quantification of intracellular ROS level [25]. DCF fluorescence was quantified as described [44] with minor modifications. Caco-2 cells were seeded in triplicate into 96-well plates at 5 × 10^4^ cells per well (200 µL/well) and grown overnight. On the subsequent day, the cells were co-treated with 100 µL of 50 µM H_2_DCF-DA (Sigma-Aldrich) and the extracts diluted 1:200 in Hank’s balanced salt solution (HBSS, Pan Biotech, Aidenbach, Germany) at 37 °C for 20 min. In each experiment, an antioxidant positive control using 20 µM quercetin (Sigma-Aldrich) was included. The cells were then washed twice with 200 µL HBSS and treated with 100 µL of the free-radical-generating compound AAPH (500 µM in HBSS; Sigma-Aldrich) or with HBSS as a control. The amount of DCF formed was determined by measuring with a POLARstar Omega microplate reader (BMG Labtech) in fluorescence mode at 485 nm excitation and 530 nm emission wavelengths immediately after the addition of AAPH and after 15, 30, 60, and 90 min of incubation. Using Graphpad Prism version 10.1.0 (GraphPad Software, San Diego, CA, USA), the DCF fluorescence intensity was background corrected, and area under the curve (AUC) values were calculated and normalized to the starting value in each well and, additionally, to the AAPH stress control as 100% and the untreated control as 0%.

### 2.7. Detection of NO Production in RAW264.7 Cells

NO production was quantified in RAW264.7 macrophages after stimulation with LPS and treatment with the plant extracts. In this assay, nitrite (NO_2−_) molecules within the culture medium react with sulfanilamide and N-(1-naphthyl) ethylenediamine dihydrochloride (NED), forming a colored azo compound. The intensity of the color, measured spectrophotometrically, aligns with the concentration of NO_2−_, a stable oxidation product of NO [26]. Detection of NO production was evaluated using the Promega Griess Reagent System according to the manufacturer’s instructions (Promega Corporation, Fitchburg, WI, USA). Briefly, RAW264.7 macrophages were seeded in triplicate into 96-well plates with a seeding density of 8 × 10^4^ cells per well (200 µL/well) and grown overnight at 37 °C, 5% CO_2_, and humidified air. On the subsequent day, the extracts were diluted 1:200 in DMEM and the cells were treated with only DMEM as a control, with 250 mg/mL lipopolysaccharide (LPS; Sigma-Aldrich) as an activator of the NO production, or co-treated with LPS and the diluted plant extracts. In addition, quercetin (20 µM in DMEM) was used as an antioxidant positive control in each experiment. After 24 h of incubation at 37 °C, 50 µL of the sulfanilamide solution were added to 50 µL of each cell supernatant and incubated for 5–10 min at room temperature. Then, 50 µL of the N-1-naphthylethylenediamine dihydrochloride (NED) solution was added. After an incubation time of 5–10 min, the amount of NO_2−_ was quantified by measuring the absorbance at 548 nm with a POLARstar Omega microplate reader (BMG Labtech). Results were normalized to the LPS stress control.

### 2.8. Determination of Cell Viability

To exclude cytotoxic extracts from the intracellular ROS and the NO production assays, a cell viability test with resazurin was conducted in the same cells after each assay. The test substances were aspirated and 50 µM resazurin sodium salt (Sigma-Aldrich) in growth medium was added to the cells (100 µL/well). After 90 min of incubation at 37 °C, the amount of resorufin formed was quantified with a POLARstar Omega microplate reader (BMG Labtech) in fluorescence mode at 544 nm excitation and 590 nm emission wavelengths. Results were normalized to the average of the control. A cell viability below 80% was considered cytotoxic and the corresponding plant extracts were excluded from the ROS and NO detection assays.

### 2.9. Statistical Analysis

Statistical analysis was performed using Graphpad Prism version 10.1.0. All measurements were taken in triplicate and mean values were calculated. The statistical difference among means was determined using an ordinary one-way ANOVA and Dunnett’s multiple comparison test. Spearman’s rank correlation coefficient (r_s_) was determined, considering the non-normal distribution of the mean values. Figures were prepared using CorelDRAW 2019 (Corel Corporation, Ottawa, ON, Canada).

## 3. Results

The 375 plant extracts were analyzed for their antioxidant activity using cell-free (TPC and FRAP), as well as cell-based, methods (ROS and NO detection).

### 3.1. TPC and FRAP Values of Plant Extracts under Study

**Figure 1:**
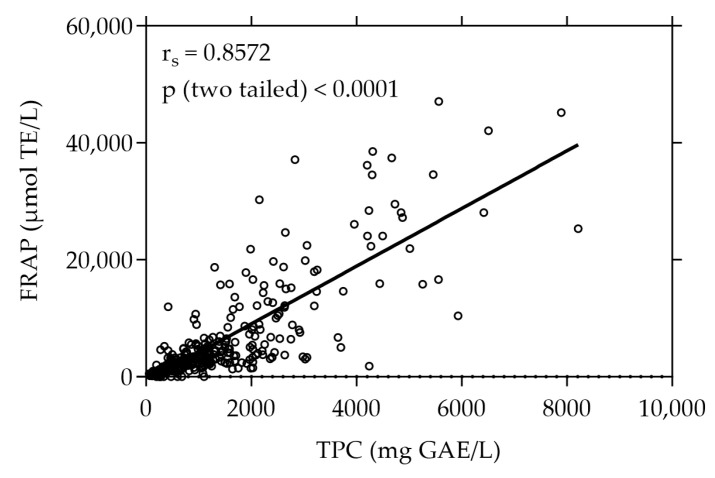
Correlation between FRAP and TPC of all plant extracts under study (n = 375). The results are expressed as µmol TE per liter and mg GAE per liter, respectively. Each plotted dot represents the mean value of 3 replicates.

The plant extracts were first screened for their total phenolic content (TPC) and antioxidant capacity determined by their reducing power (FRAP). Figure 1 shows the TPC and FRAP values of the 375 screened plant extracts. With a value of 8211.31 mg GAE/L the white-flowered oregano (*Origanum vulgare*) extract exhibited the highest TPC, whereas the strawberry leaf (*Fragaria × ananassa* “*Elianny*”) extract showed the highest FRAP value of 47,076.92 µmol TE/L. The plant extract with the highest values of both TPC (7892.86 mg GAE/L) and FRAP (45,190.48 µmol TE/L) was the pink-flowered oregano (*Origanum vulgare*) extract. The TPC and FRAP values of all 375 plant extracts are listed in Supplementary Table S1.

A strong positive correlation was observed between the total phenolic content and the antioxidant capacity expressed as FRAP, evidenced by a high Spearman correlation coefficient (r_s_) of 0.8572 (*p* < 0.0001).

### 3.2. Inhibition of Cellular ROS and NO by the Plant Extracts

**Figure 2:**
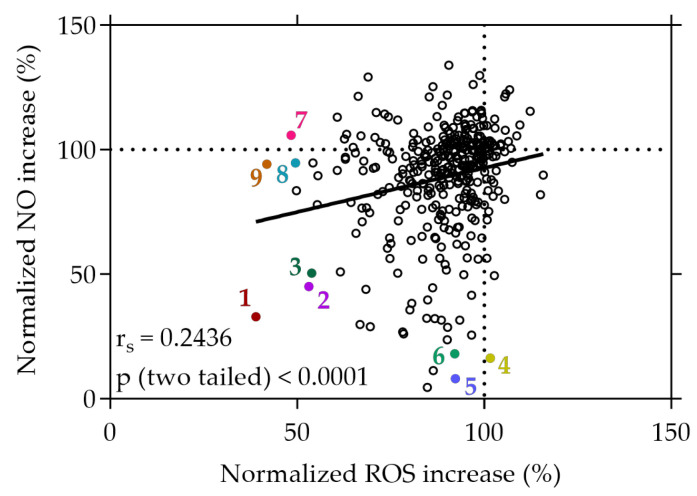
Correlation between normalized NO and ROS increase in %. Caco-2 or RAW264.7 cells were grown in 96-well plates overnight (5 × 10^4^ cells/well or 8 × 10^4^ cells/well), and then treated with the plant extracts for 20 min or with the extracts and the stressor LPS for 24 h. ROS or NO generation was determined using the cell-permeant reagent H_2_DCF-DA after AAPH-induced stress or the Griess assay, respectively. Each plotted dot represents the mean value of 3 replicates; n = 358. 1: strawberry leaf extract, 2: raspberry leaf extract, 3: wild geranium extract, 4: white cabbage extract, 5: white cabbage “Braunschweiger” extract, 6: sunchoke extract, 7: mirabelle plum extract, 8: jostaberry extract, 9: rose blossom extract.

Additional to the target-based, cell-free assay, the plant extract library was screened for candidates with potent inhibitory effects on ROS production in stressed Caco-2 cells using the cell-permeable probe H_2_DCF-DA. As displayed in Figure 2, a 20 min treatment with several of the extracts resulted in a reduction in ROS levels under stress conditions, relative to the AAPH stress control. Out of 375 tested extracts, 3 extracts were excluded due to significant cytotoxic effects, 116 extracts significantly reduced the production of ROS by Caco-2 cells, while no extract led to a significant increase in ROS levels. Notably, from these 47 hits, 34 extracts reduced the stress-induced ROS production by at least 30% (see Supplementary Table S1). In contrast, the antioxidant positive control quercetin exhibited an average reduction in ROS levels to 3.58% ± 7.45%, compared to the stress control.

Moreover, a parallel screening using RAW264.7 cells was conducted to determine the extracts’ capability to inhibit the production of the RNS NO. Among the tested extracts, 15 were excluded due to significant cytotoxicity, 148 extracts resulted in a significant decrease in NO levels, and 40 extracts significantly increased NO levels, compared to the stress control; 57 extracts were found to decrease NO levels in RAW264.7 cells by at least 30% (see Supplementary Table S1). For comparison, the antioxidant positive control quercetin decreased NO levels to 66.48% ± 17.43% on average, compared to the stress control.

The three most effective plant extracts that exhibited strong inhibitory effects on both NO and ROS (normalized NO and ROS increase < 60%), or exclusively on either NO (normalized ROS increase > 90%) or ROS (normalized NO increase > 90%), are given in Figure 2. Among all the plant extracts screened from the library, the strawberry leaf (*Fragaria × ananassa* “*Elianny*”, 1) extract exhibited the most substantial reduction of NO and ROS increase under stress conditions, with levels declining to 32.96% and 38.98%, respectively, relative to the stress control. Following the strawberry leaf extract, the raspberry leaf (*Rubus idaeus*, 2) extract (45.06% and 53.11%) and wild geranium (*Geranium maculatum*, 3) extract (50.48% and 53.88%) showed successive reductions in both NO and ROS levels under stress conditions.

Distinctively, certain extracts demonstrated a selective capacity to reduce NO levels without concurrent effects on ROS. In particular, the white cabbage “Braunschweiger” (*Brassica oleracea capitata var. alba* “*Braunschweiger*”, 5) extract exhibited a remarkable reduction of NO levels to 8.03%, accompanied by only a minimal change in ROS levels (92.31%). Similarly, cell treatment with the sunchoke (*Helianthus tuberosus*, 6) extract initiated a significant decrease in NO levels to 16.32%, coupled with a neglectable change of intracellular ROS production to 92.15% compared to the stress control. Conversely, some extracts displayed a distinct ability to solely diminish ROS generation without impacting NO levels. For instance, treatment of the cells with the rose blossom (*Rosa*, 9) extract and the mirabelle plum (*Prunus domestica* subsp. *Syriaca*, 7) extract under stress conditions decreased ROS levels to 41.86% and 48.36%, respectively, while displaying no significant effect on cellular NO levels (94.14% and 105.76%). The data imply a significant selectivity among certain plant extracts, favoring either the reduction of NO, ROS, or both simultaneously. These findings suggest that, following cell treatment under stress conditions, specific plant extracts demonstrate substantial selectivity, displaying preferences for either reducing NO levels or ROS levels, or simultaneously decreasing both.

A correlation was observed between the cellular decrease in NO production and the concurrent reduction of intracellular ROS levels, albeit displaying a weak positive relationship, indicated by an r_s_ value of 0.2436 (*p* < 0.0001), as depicted in Figure 2. This correlation suggests a subtle association between the decrease in NO and ROS levels induced by the plant extracts within the cellular context.

### 3.3. Moderate Correlations Found between Cell-Free and Cell-Based Antioxidant Assays

**Figure 3:**
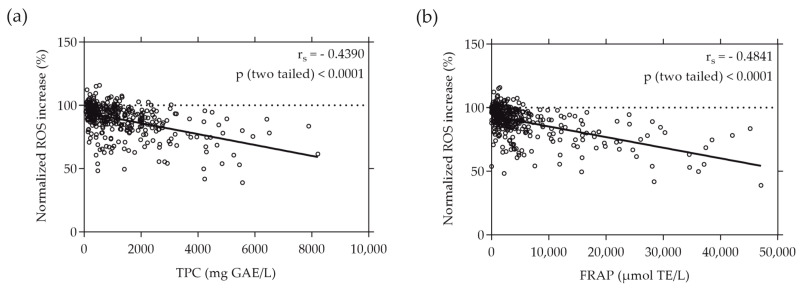
Correlation between normalized intracellular ROS increase in % and TPC (**a**) or FRAP (**b**) values of the plant extracts. TPC and FRAP values are expressed as µmol TE per liter and mg GAE per liter, respectively. Caco-2 cells were grown in 96-well plates overnight (5 × 10^4^ cells/well), and then treated with the plant extracts for 20 min. ROS increase was determined using the cell-permeant reagent H_2_DCF-DA after AAPH-induced oxidative stress. Each plotted dot represents the mean value of 3 replicates; n = 372.

When plotting the normalized ROS increase induced by the individual plant extracts against their TPC and FRAP values, a moderate correlation was revealed. The ROS increase relative to the AAPH stress negatively correlated with the total phenolic content (depicted in Figure 3a) and the reducing antioxidant power, expressed as FRAP (illustrated in Figure 3b), of the plant extracts. This negative correlation was evidenced by r_s_ values of −0.4390 and −0.4841 (*p* < 0.0001), respectively. Notably, the intracellular ROS values measured in Caco-2 cells exhibited almost identical correlation with both the TPC and FRAP values, emphasizing the consistency of this relationship.

**Figure 4:**
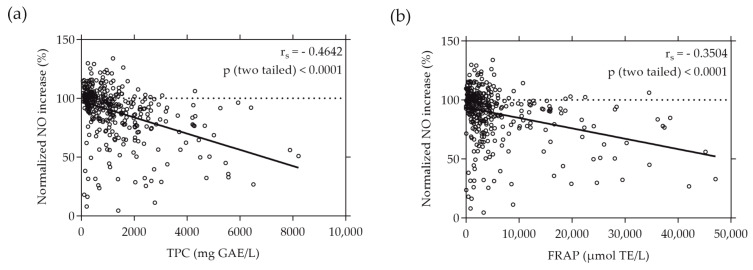
Correlation between normalized NO increase in % and TPC (**a**) or FRAP (**b**) values of the plant extracts. TPC and FRAP values are expressed as µmol TE per liter and mg GAE per liter, respectively. RAW264.7 cells were grown in 96-well plates overnight (8 × 10^4^ cells/well), and then treated with the plant extracts and LPS for 24 h. NO levels were quantified using the Griess assay. Each plotted dot represents the mean value of 3 replicates; n = 360.

Similarly, a moderate correlation was found when plotting the normalized NO increase caused by the individual plant extracts against their TPC and FRAP values, as depicted in Figure 4. The r_s_ values of −0.4642 and −0.3504 (*p* < 0.0001) indicate a moderate negative correlation between the increase in NO production by RAW264.7 cells and the total phenolic content and reducing antioxidant potential represented by FRAP, respectively.

The data presented in Figure 3 and Figure 4 indicate that extracts with the highest TPC and FRAP values did not consistently demonstrate the most substantial inhibition of NO and ROS production. Notably, among the extracts, only the strawberry leaf (*Fragaria × ananassa* “*Elianny*”) extract, characterized by the highest FRAP value, also displayed the strongest inhibitory effect on ROS and NO generation in cellular environments.

## 4. Discussion

In this study, we analyzed a comprehensive novel plant extract library comprising 375 individual extracts derived from raw plant materials predominantly collected in Upper Austria. These plants were carefully selected on the basis of non-toxicity profiles and a history of safe use for at least one part of the plant. After collection, the raw materials underwent immediate processing and extraction. Our screening process included both cell-free methods and cell-based assays to evaluate the potent antioxidant capacity of these extracts. This multi-faceted approach aimed to provide a thorough assessment of the antioxidant potential of the bioactive compounds in the extracts.

The first step involved characterizing the extracts based on their total phenolic content, a metric often associated with antioxidant capacity. Phenolic compounds, known for their antioxidant properties, utilize the hydroxyl group on the benzene ring to donate hydrogen atoms. This important mechanism neutralizes free radicals, thereby reducing oxidative stress in biological systems [45]. Additionally, we used the FRAP assay to determine the reducing power of each extract. Among the tested extracts, the white-flowered oregano (*Origanum vulgare*), strawberry leaf (*Fragaria × ananassa* “*Elianny*”), and pink-flowered oregano (*Origanum vulgare*) extracts showed the highest TPC and FRAP values. Oregano (*Origanum vulgare*) extracts and essential oils have previously been described to contain high levels of phenolic compounds, with carvacrol being the main polyphenol [46,47]. Strawberry (*Fragaria × ananassa*) leaves also contain high amounts of polyphenols and are described to exhibit antioxidant and radical scavenging activities measured by various cell-free assays [48]. It should be noted that comparing absolute values for TPC and antioxidant capacities among extracts from the same raw materials but from different studies often presents challenges due to the influence of various factors on the chemical composition and bioactivity of the extracts. Parameters such as geographical location, harvesting time, prevailing climate conditions, and extraction parameters can introduce considerable variability, making a direct comparison of different study results difficult [49].

To discern the potential relationship between the phenolic content and reducing the antioxidant capacity of our plant extract library, we analyzed the correlation of the TPC and FRAP values. A high positive correlation between TPC and FRAP values among the tested extracts was revealed. This correlation suggests that a higher phenolic content within the extracts significantly corresponds to increased reducing antioxidant capacity as assessed by the FRAP assay, highlighting the contribution of phenolic compounds to the overall antioxidant potential of the extracts. The observed positive correlation between these parameters was not unexpected given the fundamental similarities in the underlying principles and chemistry of the Folin–Ciocalteu TPC assay and the FRAP assay. Both methods focus on the ability of the test substance to exhibit reducing power. In the TPC assay, phenolic compounds reduce the Folin–Ciocalteu reagent in an alkaline environment, resulting in the formation of blue chromophores due to the reduction of the reagent’s metal oxides. Similarly, the FRAP assay quantifies the intensity of the color formed from the product of the reduction of the ferric-tripyridyltriazine complex catalyzed by antioxidants in the sample [50]. However, the TPC assay is susceptible to interference from non-phenolic compounds present in the plant extracts, including reducing sugars, amino acids, or proteins, thereby affecting accuracy and resulting in TPC overestimation [19]. Therefore, both assays essentially measure a similar parameter. Nonetheless, a high positive correlation between the TPC and FRAP values emphasizes the antioxidant potential of the plant extracts while reinforcing the reliability of their assessment. TPC and FRAP measurements are often used as an initial approach for swiftly evaluating the antioxidant activity of plant-derived extracts [51,52,53,54]. Recently, Priyanthi and Sivakanesan reported a similar correlation coefficient (r = 0.8534) between TPC and FRAP across extracts of various rice varieties [51]. Similarly, in another study, Diep et al. demonstrated an even stronger correlation between TPC and FRAP among extracts derived from different tamarillo cultivars, affirming the robust association between these parameters across different plant sources [52].

Additionally, we conducted a more detailed analysis of the antioxidant capabilities of the extracts using cellular systems to address limitations of target-based, cell-free methods. The lack of a complex cellular environment in these methods hinders the ability to capture crucial aspects such as cellular uptake, metabolism, compound transformation, and the intricate interactions and signaling within cells. Therefore, the assessment of the antioxidant capacity might be limited. This underscores the need for screening in cellular systems, in addition to target-based readouts to provide a more comprehensive understanding of antioxidant behavior in living organisms [21]. Our plant extract library was screened for potent inhibitory activity on ROS and NO production under stress conditions. To our knowledge, no study has previously focused on a comprehensive assessment of the ability of plant-derived extracts to inhibit both ROS and NO production within cells. During the screen of the extract library, we observed diverse behaviors in some of the extracts’ effects on ROS and NO levels. While treatment with certain extracts led to an inhibition of both ROS and NO generation, others displayed strong reducing effects solely on either ROS or NO levels. This discrepancy in the modulation by the extracts highlights the diversity of their biological activities and suggests the presence of different main bioactive components or mechanisms within the extracts. Among all the plant extracts screened in the library, the strawberry leaf extract (*Fragaria × ananassa*) showed the most significant reduction in both ROS and NO accumulation, under stress conditions, closely followed by the raspberry leaf (*Rubus idaeus*) and wild geranium (*Geranium maculatum*) extracts. Strawberry and raspberry leaves contain high amounts of polyphenols, and both are described to possess antioxidant and radical scavenging properties as detected via cell-free assay techniques (e.g., ORAC and DPPH radical assay) [48,55]. To our current knowledge, there are only limited studies available describing the effects of strawberry and raspberry leaf extracts on ROS and NO levels within cells. Liberal et al. demonstrated remarkable NO scavenging activity attributed to the leaf extract of *Fragaria vesca*. The study suggested that the primary polyphenols in the extract, specifically the ellagitannin sanguiin H-6/agrimoniin isomer, along with glucuronide derivatives of quercetin and kaempferol, contributed significantly to this observed activity [56]. Certain *Geranium* species are described to be rich sources of phenolic compounds; however, the antioxidant profile of *Geranium maculatum* extract has not been described so far [57]. Here, we provide evidence that the extract from *Geranium maculatum* can induce a strong reduction of both NO and ROS levels, in cells under stress conditions. Furthermore, in our screen, treatment with the white cabbage extract (*Brassica oleracea convar. capitata var. alba L*) and the sunchoke extract (*Helianthus tuberosus*) notably suppressed NO production and secretion while displaying neglectable effects on ROS levels. Although both plants have a history of traditional medicinal use and are described to exhibit some bioactive properties, little is known about their ability to modulate NO and ROS levels [58,59,60]. Similarly, to our current knowledge, there are no studies available on the antioxidant potential of mirabelle plum extract (*Prunus domestica* subsp. *Syriaca*) and jostaberry extract (*Ribes × nidigrolaria*), which showed strong ROS scavenging activity but no effect on NO levels in our plant extract library screen.

Our study revealed a subtle positive correlation between ROS and NO levels following extract treatments under stress conditions. This observation suggests a potential underlying relationship between the effects of the extracts on these key cellular signaling molecules. So far, only few studies have focused on the concurrent evaluation of ROS and NO impacts induced by plant extracts. Examples include extracts prepared from the leaves of *Pseuderanthemum palatiferum* [61] and the rhizomes of *Curcuma longa* L. [62], both of which have demonstrated the ability to attenuate ROS and NO under stress conditions.

When comparing the cell-free antioxidant assays with these cellular assays, we observed a moderate negative correlation between an ROS and NO increase and TPC and FRAP values, suggesting a visible tendency or trend between these parameters. However, the correlation was not consistently proportional or direct across all samples tested, implying that factors beyond the phenolic content or reducing power might influence ROS and NO generation. Notably, the plant extracts with the highest TPC and FRAP values did not necessarily exhibit the strongest inhibitory effects on ROS and NO production. Only the strawberry leaf (*Fragaria × ananassa* “*Elianny*”) extract, which showed the highest FRAP but not TPC value, also reduced the ROS and NO levels the strongest. While TPC and FRAP assays provide valuable information on the reducing capacity of antioxidants in a controlled environment, the ROS assay reflects the practical radical scavenging ability within a cellular system. This distinction highlights the difference between measuring the potential of antioxidants to donate electrons (as seen in TPC and FRAP assays) and their actual efficacy in scavenging free radicals and mitigating oxidative stress in a biological context (as assessed by ROS assays) [21]. The observed moderate negative correlation between NO levels and TPC values indicates that phenolic compounds within the extracts may play a role in influencing NO levels, implying a possible interplay between antioxidant components and the regulation of NO production. On the other hand, the weaker negative correlation between NO levels and FRAP values suggests a less definitive relationship between total reducing power as measured by FRAP and the modulation of NO levels. This suggests that, while FRAP provides insight into the reducing power of antioxidants, it may not influence or reflect the modulation of NO levels as strongly as TPC. These different correlations highlight the multifaceted nature of the bioactivity of plant extracts. In recent years, Sun et al. discovered a discrepancy in the inhibitory effect of individual phenolic compounds or extracts from sweet potato leaves on intracellular ROS levels compared to their antioxidant activity as measured by the oxygen radical absorbance capacity (ORAC) assay and automated photo-chemiluminescence (PCL) assay [63]. Sun and colleagues postulated that the preventive effect of sweet potato leaf polyphenols on ROS generation in human LO2 hepatocytes under H_2_O_2_-induced stress may be mediated through the regulation of oxidative-stress-related gene expression rather than a direct antioxidant effect. Conversely, Xiong et al. reported that the TPC values of selected common edible flower extracts strongly correlated with their antioxidant capacity assessed by several cell-free assays, including the FRAP and ORAC assays, and that the results are almost consistent with the extracts’ ability to scavenge intracellular ROS [64]. Other studies have similarly indicated a direct connection between target-based cell-free techniques assessing the inhibition of radicals such as hydroxyl, superoxide, and 2,2-diphenyl-1-picrylhydrazyl (DPPH) radicals, and the ability of plant extracts to scavenge ROS and NO within cellular environments [65,66,67,68].

In conclusion, the comprehensive evaluation of the antioxidant potential of plant extracts requires a careful and multifaceted approach that integrates various assay readouts and screening techniques including both fast target-based in vitro and more complex cellular approaches. Cell-free methods provide quantifiable measures of antioxidant capacity under controlled, isolated conditions, elucidating the chemical antioxidant properties of the extracts. On the other hand, cellular assays evaluate the efficacy of the extracts in a live cell environment, thereby increasing biological relevance. The findings of the plant extract library screen imply the promising potential of certain plant extracts for further investigation as potential components of functional foods and dietary supplements. The strawberry leaf (*Fragaria × ananassa* “*Elianny*”) extract, especially, as well as the lesser studied raspberry leaf (*Rubus idaeus*) and wild geranium (*Geranium maculatum*) extracts, emerged as great antioxidant candidates. Future research should focus on the investigation of specific bioactive compounds within the extracts and their individual contributions to antioxidant activity. Additionally, exploring the effects of the extracts on various cellular pathways involved in oxidative and nitrosative stress responses may reveal novel therapeutic potentials.

## Data Availability

The data are available from the authors upon request.

## Supplementary Materials

The following supporting information can be downloaded at: https://www.mdpi.com/article/10.3390/antiox13030297/s1. Table S1: Plant extract library database containing the name, genus/species, cite, and date of collection and plant parts used of the plant raw materials, as well as the results of the TPC, FRAP, NO, and ROS detection assays. Results are depicted as mean values (n = 3).
